# Sustainable Development under Population Pressure: Lessons from Developed Land Consumption in the Conterminous U.S.

**DOI:** 10.1371/journal.pone.0119675

**Published:** 2015-03-25

**Authors:** George Grekousis, Giorgos Mountrakis

**Affiliations:** State University of New York, College of Environmental Science and Forestry, Department of Environmental Resources Engineering, 402 Baker Laboratory, 1 Forestry Drive, Syracuse, New York, 13210, United States of America; University of Gävle, SWEDEN

## Abstract

Population growth will result in a significant anthropogenic environmental change worldwide through increases in developed land (DL) consumption. DL consumption is an important environmental and socioeconomic process affecting humans and ecosystems. Attention has been given to DL modeling inside highly populated cities. However, modeling DL consumption should expand to non-metropolitan areas where arguably the environmental consequences are more significant. Here, we study all counties within the conterminous U.S. and based on satellite-derived product (National Land Cover Dataset 2001) we calculate the associated DL for each county. By using county population data from the 2000 census we present a comparative study on DL consumption and we propose a model linking population with expected DL consumption. Results indicate distinct geographic patterns of comparatively low and high consuming counties moving from east to west. We also demonstrate that the relationship of DL consumption with population is mostly linear, altering the notion that expected population growth will have lower DL consumption if added in counties with larger population. Added DL consumption is independent of a county’s starting population and only dependent on whether the county belongs to a Metropolitan Statistical Area (MSA). In the overlapping MSA and non-MSA population range there is also a constant DL efficiency gain of approximately 20km^2^ for a given population for MSA counties which suggests that transitioning from rural to urban counties has significantly higher benefits in lower populations. In addition, we analyze the socioeconomic composition of counties with extremely high or low DL consumption. High DL consumption counties have statistically lower Black/African American population, higher poverty rate and lower income per capita than average in both NMSA and MSA counties. Our analysis offers a baseline to investigate further land consumption strategies in anticipation of growing population pressures.

## Introduction

Population increase, mobility and urban growth have and will continue to induce significant environmental changes [1,2]. It is estimated that between one third and one half of the planet's land surface has been transformed by human activities [3]. These land cover changes are at the center of the human-environmental sciences [4]. The impacts of these changes can be observed at various scales throughout the globe and include forest loss, atmospheric composition alterations, regional temperature fluctuations and precipitation variability [5,6,7,8]. A major component of these changes is land development for residential, commercial or infrastructural needs [9]. Known as Developed Land (DL), this is defined as the horizontal spatial footprint containing constructed materials (e.g. buildings, roads, parking lots, and airports) [10]. DL does not solely refer to the land inside an urban centre and it is not bounded by population sizes (e.g., cities, towns or villages).

Although DL area occupies less than 3% of earth's surface, land transition from other land cover types to DL is regarded as one of the most irreversible human impacts on the global biosphere and is contradictory to sustainable development [9,11]. DL area increase to support cities and population substantially alters ecosystem functions with significant consequences for humans, biodiversity and natural resources. For example, forests and croplands have been the largest source of land transitioned to DL with significant ecological impacts [11]. DL land conversion also leads to other environmental changes such as increased minimum temperatures, alterations in precipitation patterns and urban heat island effects [9]. Furthermore, increases in urban population and growth of surrounding DL areas could lead to higher traffic congestion and concentration for atmospheric pollutants [11]. For these reasons, DL area increase is an integral consideration in planning and has been recently included in political discussions regarding sustainable development [12–14]. DL area has been used as a key characteristic to build indicators to monitor sustainable development and track urban and suburban ecosystems changes. For example, the percentage of DL in total land area has been used as one of the 155 sustainable development indicators adopted by the European Union [15]. DL has also been defined as a component of the ecological footprint indicator [16] and is one of the 108 indicators defined to describe the condition and use of the USA's ecosystems [17].

At the global scale, population increase and urban growth cause significant pressure on land availability turning land to a scare resource [18]. Today, the urban extent is increasing twice as fast as the population itself [19]. Furthermore, models suggest that urban land area will nearly double between 2000 and 2030 in North America and Western Europe, and increase 600% in Africa [1,19]. Another study predicts that in the same period, the world's population living in cities larger than 100,000 people will increase by 72%, while DL area will increase by 175% [20]. Additionally, in 22 countries of the EU urban land area is expected to increase by 28% from 2000 to 2020 [21]. While DL areas remain a small portion when compared to the entire landscape, the urban land is expected to double in the next 20 years [1].

This DL area explosion in such a short timeframe presents an emerging need for further analysis of DL consumption and associated efficiency in land resources savings in order to comply with sustainable development declarations. Creation of associated DL efficiency metrics is essential for sustainable development planning. In this manuscript we focus on the conterminous United States (U.S.), where remarkable DL changes have been recorded in the past and are projected for the future. While DL area accounted for approximately 235,000 km^2^ and 3% of the U.S. land area in 1973, there was a 33% increase and by the year 2000 DL area accounted for 313,000 km^2^ (4% of total U.S. land) with a similar increase in population of 38% [22]. The DL area increase has been estimated as being even higher between 1982 and 2007 at 57%. [23]. A 30% population growth, which is anticipated in the U.S. between 2003 and 2030, is expected to result in a 51% increase in DL area consumption [24,25]. Other studies suggest that by the year 2030 18 million hectares of private rural land inside forested watersheds in the U.S. will be converted to residential developments [26] and that additional housing units in and within 1km of protected areas will increase at an average rate of 52% a year from 2000 [27]. In other estimations, significant forest loss due to urban development is expected by 2062 with an estimated forest to DL conversion of nearly 50 million acres [28]. This acceleration of U.S. urban growth has contributed to human well-being, but also has posed challenges for ecosystem services [22].

As DL area increase will partially take place inside cities, theories have been developed relating city scaling, population increase and DL expansion [29,30]. A power relationship between per capita infrastructure and population has been identified indicating efficiency gains for higher populations [31]. As cities increase in population the per capita space needs for transportation and residential living decrease [32]. However, modeling DL consumption should not be limited to city boundaries and should expand to rural areas, where arguably the environmental consequences make it more significant. For example, the aforementioned analysis using power relationships, deals with phenomena within cities but says little about cities with boundaries that are hard to define where one city ends and another begins [30].

In fact, the urban landscape of the future will be a mixture of continuous urban spaces with varying densities and DL consumption will have a significant effect beyond the edges of traditionally defined urban areas [33, 34]. Although attention has been given to DL modeling inside cities, as presented in the works above, there is a clear need to expand models across the entire landscape.

In addition, DL consumption, when contrasted exclusively with population data, does not provide a complete picture, as numerous and complex interactions exist between socioeconomic (SE) characteristics and DL. From the wide range of SE characteristics, for example demographic variables, annual growth in GDP per capita, capital flows, agricultural vs industrial economy, we focus on key demographic variables including race, education, poverty and income. It is now acknowledged that the analysis of the demographic characteristics and behaviors of people and the identification of subpopulations that contribute at a higher or lower degree to sustainable development should be put at the center of the research agenda [35]. Linking of socioeconomic variables with raster data may lead to a more comprehensive analysis of the human environment and its impact on land resources and land cover dynamics [36].

Concluding, from the land consumption perspective there is no national comparative study that i) examines the transition from rural to urban, ii) provides a relative ranking of counties/states based on their DL consumption and iii) analyzes the socioeconomic composition of high and low DL consumption counties. Creating a predictive and qualitative framework associated with specific metrics is acknowledged highly as important in understanding city dynamics in the context of sustainable development and human prosperity [37].

The objectives of our work are to fill these gaps and: i) to provide the first wall-to-wall national DL consumption comparative analysis, and ii) to investigate the socioeconomic composition of high and low DL consumption counties. We provide a relative ranking of counties based on their DL consumption that identifies counties and states that over or under perform when compared with the median expected DL area consumption for their given population. A model fitting process further enhances our findings by identifying the per capita DL area benefit of transitioning from a rural to an urban county of similar population. Socioeconomic analysis is based on variables related to race, education, income and poverty.

Our analysis uses the National Land Cover Dataset 2001 (NLCD) to calculate the associated DL footprint for all counties within the conterminous U.S. DL is one of the 16 land cover classes of the NLCD 2001. As DL area is not equally distributed across administrative boundaries or population, we calculate a population adjusted DL to rank and compare counties with similar populations. County boundaries and population estimates are gathered from the 2000 Census. Population adjusted DL aims at locating counties with similar populations but with different levels of DL area. If county A uses less DL area to serve a population compared with another county B with similar population then we consider county A more efficient.

The efficiency term relates to DL consumption per capita. It should not be automatically assumed that it is a positive attribute. On one hand, high efficiency, expressed as low DL area consumption per capita, offers environmental benefits. On the other hand, that same low DL consumption may be a result of limited infrastructure and services associated with human well-being. Efficiency is only related to land consumption.

Depending on the ranking position of each county we mark them as having high, medium, or low efficiency. Power and linear fit models are applied to model DL consumption with population and to identify developed land gains from transitioning from non-metropolitan statistical areas (NMSA) to metropolitan statistical areas (MSA) counties with similar population (MSA and NMSA are defined in section 2.1). The linear models may be used for estimating DL area for population change scenarios or population estimation if DL area size can be extracted via in situ observations (e.g. satellite imagery). Our results reveal for the first time the DL consumption benefit (infrastructure efficiency gain) of transitioning from a rural to an urban county (20km^2^). A comparative guide is provided identifying counties and states that can be labeled as having low or high efficiency in terms of developed land consumption. Further analysis took place to examine the socioeconomic (SE) characteristics of counties with extreme DL behavior. Significant variability in these characteristics can lead to further understanding of DL consumption patterns. The outcomes of our study may be used for a more sustainable planning by supporting planners and policy makers for applying smart urban growth or containment policies more efficiently. The distribution of added population should be made by balancing DL consumption and our study gives practical guidelines through the proposed linear model fits.

## Materials and Methods

To analyze DL area consumption over the conterminous US we follow a four step procedure. First, we extract the DL area for each county using the NLCD 2000 product. Second, we adjust DL by population and compare similarly populated counties. Third, we progressively group counties with similar population, identify the median DL for that group, and then across multiple groups use linear and power fits to model expected median DL consumption. Conclusions on efficiency are drawn based on model fits and county relative ranking. Comparisons on DL consumption also take place between MSA and NMSA counties. Fourth, the socioeconomic composition of high and low DL consumption is analyzed.

### 2.1 Data sources and associated accuracy

Raster data expressing DL distribution, vector data representing county boundaries and county population data are used in this analysis. The use of remotely sensed imagery offers great potential to delineate DL consumption at large scales. In this study, NLCD 2001 produced by the Multi-Resolution Land Characteristics Consortium [38], is used to extract developed land (DL) area, at a nominal pixel scale of 30m × 30m with a 5 pixel minimum mapping unit, for each county. Developed land is characterized by a high percentage (more than 30%) of constructed materials. The overall accuracy for the NLCD 2001 in the conterminous US for Anderson Level I thematic classification based on the mode definition of agreement is 85.3% [10]. National accuracy results have been assessed on 10 geographic regions with user’s and producer’s accuracy for DL class (Level I, NLCD 2001 Class 20, mode definition) ranging from 57.0–92.9% and 43.0–87.0%, respectively (S1 Table, S1 Fig.). Since our method aggregates DL pixels at the county level our expected errors can be expressed as the difference between commission and omission errors, which strongly relate to user’s and producer’s accuracy. “S1 Table” indicates a consistent overestimation of DL throughout the 10 geographic regions, with the exception of region 4, where DL is underestimated. Visual inspection of “S1 Fig.” does not suggest a significant geographic bias for region 4.

Population and SE data were obtained from the National Historical Geographic Information System (NHGIS) referring to the year 2000 at the county level [39]. A detailed report regarding the accuracy of the census data can be found in [40] and [41]. We calculated White, and Black/African American population percentages per county. Race comes from census at a 100% count. As far as educational attainment we created a variable named Higher Education. It is the percentage of people having high school diploma and higher and was calculated by the aggregation of all categories including 'High School Graduate', 'Some college, No degree', 'Associate Degree', 'Bachelor's Degree', Master's Degree', 'Professional School Degree', and ‘Doctorate Degree’. We also included poverty status in 1999 as the percentage of households being below the poverty level and we name this variable Poverty. Poverty is calculated as the ratio of households below poverty level to total households of a county. Per Capita Income was retrieved directly from census bureau databases without any other modification. Education level, income and poverty are based on responses from a sample population. As a result, estimates may vary from the actual values but there is a statistical significance at the 90% confidence level.

We use counties as our spatial units to encapsulate the vague distinction of where a city ends and another begins, a condition already observed in many parts of the world [30]. Counties are analyzed separately through assignment to the MSA or the NMSA group (S2 Fig.). A MSA is defined as an area with at least one urbanized center with a population of at least 50,000 people and comprises one or more central counties containing the core and the adjacent outlying counties that have high degree of social and economic interactions [42]. All the remaining counties (including Micropolitan Statistical Areas) are classified in this study as NMSA. Following the example of other works [9, 43] we study separately MSA (n = 842) from NMSA (m = 2,267) counties. Geographic data of counties, MSA and NMSA areas refer to the 2003 cartographic boundaries created by US Census Bureau [44].

### 2.2 DL estimation per county

A vector file representing county boundaries was overlaid on the NLCD 2001 mosaic raster dataset in a GIS environment. Zonal statistics were applied to calculate the total pixel count for every NLCD class inside each county. For each county, the number of pixels classified as ‘developed land use’ multiplied by the pixel area produced the total developed land area for each county. The process of calculating the developed land pixels inside each county introduced a minor measurement error. For example, accuracy issues exist for pixels on the county boundaries. As pixels are square, pixels crossed by boundaries are attributed to only one of the two counties sharing the same border. Therefore, DL is calculated from all pixels that have at least 50% of their pixel area fall inside a county’s boundary. Furthermore, similar accuracy problems exist in county boundaries that separate land and water (sea, rivers or lakes). When land pixels fall outside the boundary, or lie more than 50% outside the boundary, they are not included in the county's area. These are inevitable problems when dealing with raster to vector conversions or zonal calculations and are highly dependent on the raster resolution and vector positional accuracy. Due to the small number of pixels depicting DL class that lie over boundaries compared to county size no significant bias is introduced in our analysis.

### 2.3 Population adjusted DL and county ranking

We make use of DL area as a way to assess land consumption reflecting the human settlement space inside each county for the conterminous US. As DL has a strong correlation with population (for the conterminous US, r = 0.86), it is expected that higher population will lead to increased DL consumption. In addition, DL has large variability over population (S3 Fig.). To take these into account we adjust DL with population and compare similarly populated counties.

Our analysis includes a local population adjustment by comparing each county’s DL to others of similar population. Population adjusted DL aims at locating counties with disproportionally high or low DL consumption. A ranking method is applied separately on MSA and on NMSA. To rank each county, MSA and NMSA counties are sorted by population. For the county under consideration (reference county) a group of 101 counties is produced for comparison purposes. This group contains 50 counties with the closest yet smaller population than the reference county and 50 with closest and larger population. The counties of this group are sorted again by DL value to calculate the relative ranking position of the reference county regarding DL value. The final rank reveals the relative position of reference county regarding DL inside the group of 101 counties with similar population. A higher relative ranking position of the central county reflects increased DL consumption compared to counties with similar population. The first 50 and last 50 counties of MSA and NSMA respectively are excluded as they cannot form symmetrical groups based on the 50 previous or 50 following counties (total 200 counties). These counties are regarded as outliers as they have either very small or very high population and caution should be applied not to extrapolate results in those extreme cases.

There is no DL normalization for county size since the overall correlation of DL values with county size is weak (r = 0.19). The weak trend for larger DL in larger counties, as a result for example of longer road network or different network grid size, does not affect our study. In fact it is captured and accounted for a county's DL efficiency, as for example the long road network serving farms in large agricultural rural counties. Since the motivation of our work is a national comparison it is of interest to contrast counties of similar population but different structure (e.g. low and high agricultural levels).

### 2.4 Models

Linear and power models are fitted on median DL separately for MSA and NMSA counties. The power fit model is applied, for comparison with the linear model, as recent work in highly populated cities suggests that urban properties like infrastructure, income or crime are governed by power laws [27]. These fitted models express the median efficiency model in terms of DL consumption after adjusting for population. Deviation from the model measures how each county over or under performs relative to expected DL value for its population. Counties below the linear fitted model are characterized as more efficient because they consume less DL for their population while counties above the trend line have lower efficiency.

### 2.5 Socioeconomic analysis

The examined socioeconomic characteristics included Race (White population %, Black or African American population %—for simplicity reasons we call the Black or African American category as AA), Education (Higher Education as % population with at least a High School degree), and Economic indicators (Income per capita, Poverty as % households below the poverty level). The corresponding MSA and NMSA distributions of each SE characteristic are statistically compared among three groups, the LC (Low Consumption <10% rank), HC (High Consumption >90% rank), and all counties (ALL) using the Mann-Whitney median U-test [45]. The hypothesis tested is Ho: there is no difference in median values of a certain SE characteristic distributions belonging to different DL consumption groups. For example, we use the U-test to compare the White population distribution of an LC and the HC NMSA groups. A P-value is calculated and if smaller than a reasonable significance level *a* (*a* = 0.05 / 0.01 / 0.001) there is compelling evidence to reject Ho and conclude that these two White population distributions have statistically significant differences.

## Results and Discussion

**Fig 1:**
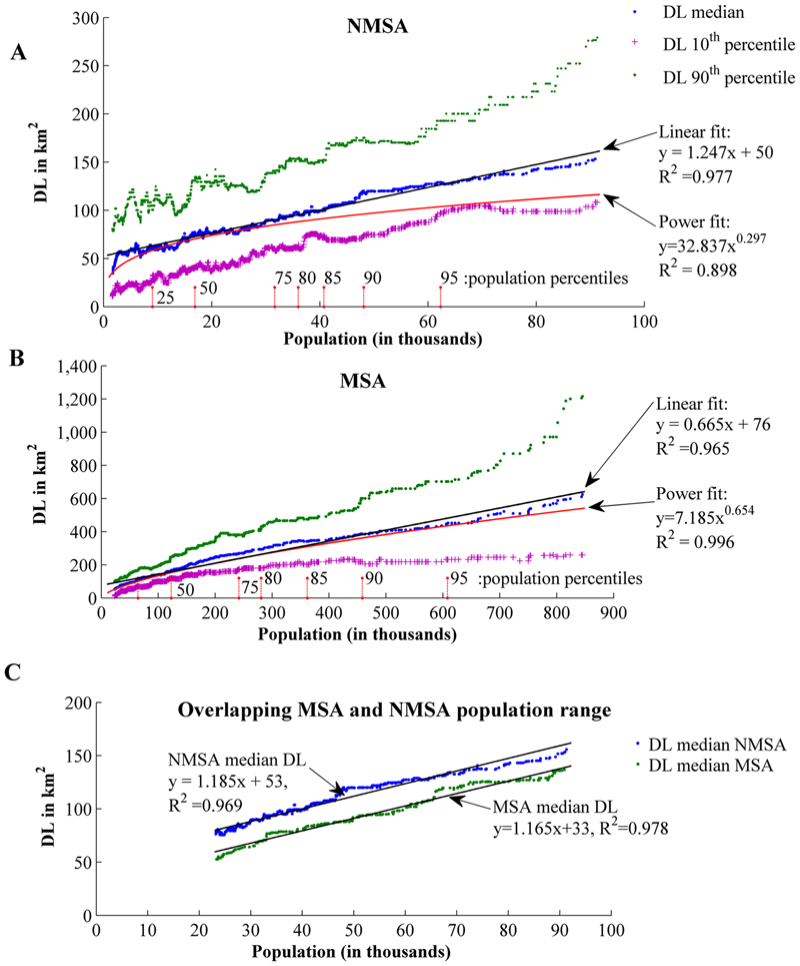
Linear and power models. Median DL, 10^th^ percentile and 90^th^ percentile DL values for the 101 counties groups for NMSA (A) and MSA (B). Population percentile distribution for each county group is also marked on X axis for Fig. 1A and 1B. NMSA counties generally have smaller populations with 9,054/16,900/31,667 for 25th percentile, median and 75th percentile, respectively. The corresponding percentiles for MSA counties are 64,350/122,934/241,423. There is an overlap of NMSA and NMSA for populations between 23,354 and 91,292 people. Direct comparison of median DL for NMSA and MSA counties in overlapping populations reveals a nearly constant additional DL consumption for NMSA counties (C).

DL area for the year 2001 for each county in the conterminous U.S. is depicted in S4 Fig. Due to the ranking process for adjusting DL with population, 200 counties are excluded from the analysis and labeled outliers. In total, 2,167 and 742 groups are created for NMSA and MSA respectively, as many as the number of counties in each case (NMSA (n = 2167) and MSA (n = 742)). For each reference county the median DL of the group it belongs to is calculated. Reference counties are plotted in population ascending order along with their corresponding median DL value calculated above (Fig. 1). Linear and power models are fitted to express the median efficiency model in terms of DL consumption.

Fig. 1A and Fig. 1B present the 101 county moving median DL value and two extreme DL percentiles. The 10^th^ percentile is associated with counties having high efficiency and the 90^th^ percentile reflects counties having low efficiency. Fig. 1C focuses on median DL values for the overlapping NMSA (n = 775) and MSA (n = 284) population range (23,354–91,292 people) (see S5 Fig. for the overlapping counties).

The linear fit model is applied to best fit NSMA and MSA median DL distribution. The linear fit for NMSA has an R^2^ = 0.977 and an R^2^ adjusted = 0.971 and the fit for MSA has an R^2^ = 0.965 and R^2^ adjusted = 0.960.The power model applied to the same dataset achieved R^2^ = 0.898 and R^2^ adjusted = 0.881 for NMSA and R^2^ = 0.996 and R^2^ adjusted = 0.992 for MSA. Our findings suggest that the previously identified non-linear relationship [29,30,34] does not extend to smaller population ranges or larger spatial units (counties). Starting with NMSA counties it is both statistically and visually evident that a linear model approximates better the expected median DL (Fig. 1A). In MSA counties (Fig. 1B), the differences between linear (R^2^ = 0.965) and power models (R^2^ = 0.996) are minor visually and statistically.

As population is likely to increase, an interesting question relates to the added DL consumption associated with population increases. The low fitting error for both NMSA and MSA counties indicates that these models may be used to calculate the expected median DL under different population scenarios. Under our linear model if the same population is added to a low population and a high population NMSA county the added DL consumption will be the same. The same holds true for MSA counties. Policy-wise, this is a significant finding as linear relationships suggest similar DL change for a given population increase, as opposed to considerable DL efficiency gains that the power law implies. In addition, slope parameter values express the added DL that one additional human consumes inside each county. Using the corresponding slopes of the NMSA and MSA linear models over the entire population range we can expect DL median demand twice as high in non-metropolitan than in metropolitan areas (MSA counties suggest a DL addition of 665 m^2^ per added person vs. 1247 m^2^ for NMSA) (Fig. 1A and Fig. 1B). The slope can also be inversely used to estimate median developed land abandonment (houses, parking lots, driveways, brownfields) if population decreases. In the latter case, the outcome of the linear fit model calculates the expected median DL area for the new population and not the actual decrease in DL value, as it is unlikely that abandoned areas will be completely converted to other land cover classes. As a result the use of the model for the inverse direction is not always straightforward and it should be considered regarding the data in hand. Developed land abandonment is an important indicator for sustainable development [46].

The projection analysis leading to DL estimation for a given population increase is based on space for time substitution. This technique, mostly used in ecological studies, assumes that spatial and temporal variations are equivalent and thus we can infer a temporal trend by studying sites of different age [47] (Text A in S1 File). The large county sample size (nearly 3000 counties) provides confidence that the use of space for time substitution is reasonable and thus we can assume a bi-directional temporal trend by studying counties of different population. Inversely, efficiency models can infer population if DL is monitored by remotely sensed imagery, thus providing an intermediate low cost population assessment between census efforts. In a broader context, as continued population growth is a global issue, these models could be useful for population estimation especially in developing counties with limited census information. Different models would be necessary to reflect regional/continental differences, for example borrowing a model from one African country with suitable census data on another with similar socio-economic structure but with inadequate census information.

### MSA and NMSA comparison in overlapping population range

MSA and NMSA counties are compared using linear fits in the overlapping 23,354 to 91,292 population range (Fig. 1C, S5 Fig.). Using exclusively this overlapping range allows us to disregard influence from high leverage points that typically appear in population extremes and offers the opportunity for decision makers to contrast counties with similar population ranges to assess different DL consumption patterns. This is especially evident in the MSA counties where the MSA population range spans from 23,354 to 845,303 while the NMSA-MSA overlapping population reaches up to 91,292.

To calculate the difference of median DL between NMSA and MSA we estimate the linear models that best fit NSMA and MSA using median DL values exclusively within this population range (Fig. 1). For MSA the linear model is y = 1.165x+33 (R^2^ = 0.978, R^2^ adjusted = 0.975) and for NMSA it is y = 1.185x+53 (R^2^ = 0.969, R^2^ adjusted = 0.965). The y values depict DL in km^2^ and the x values correspond to county population in thousands. The slopes of the NMSA and MSA models are almost identical and the difference of intercepts is the constant difference between NMSA and MSA median DL.

The fact that NMSA counties have lower efficiency, when compared to MSA counties with similar population, is not surprising. However, this is the first time this efficiency difference is quantified and surprisingly it is constant for the median values over the overlapping population range. If we compare median values, a NMSA county with similar population as a MSA county consumes an additional 20km^2^ of DL. Alternatively, an MSA county having the same DL with a NMSA county would be serving approximately 18,000 more people. In addition the 10th and 90th percentiles are more or less parallel for the NMSA showing similar behavior for the low and high population NMSA counties. On the other hand, MSA 10th and 90th percentiles are symmetrical. The deviation between 10th and 90th percentile from the median increases as population increases. This means that there is high variability in the efficiency of MSA counties-something that should be further analyzed to reveal potential hidden structure in data. The consistent advantage for MSA counties for the median values expresses DL efficiency gains from consolidation of services and infrastructure, among others. Although not directly evaluated in this research, we believe that this might be the result of MSA counties' successful adoption of state growth management programs, such as containment policies and smart growth strategies [48–51]. The objective for these programs is to reduce urban sprawl by adopting regulations, for example by clustering horizontal residential development into areas with existing substantial infrastructure[52] and/or increasing vertical sprawl on existing horizontal footprint [53]. Another possible explanation is the different spatial structure between MSA and NMSA. The increasing growth of the metropolitan areas has led to a dispersal of new services outside the main urban core, towards the outlying counties, and many of these services are clustered in concentrated locations named edge cities [54]. Most edge cities arose at the conjunction of interstate highways or urban corridors and maximize accessibility [55]. As a result, services and infrastructure (e.g. road network) are shared among several counties.

### County and State efficiency ranking

**Fig 2:**
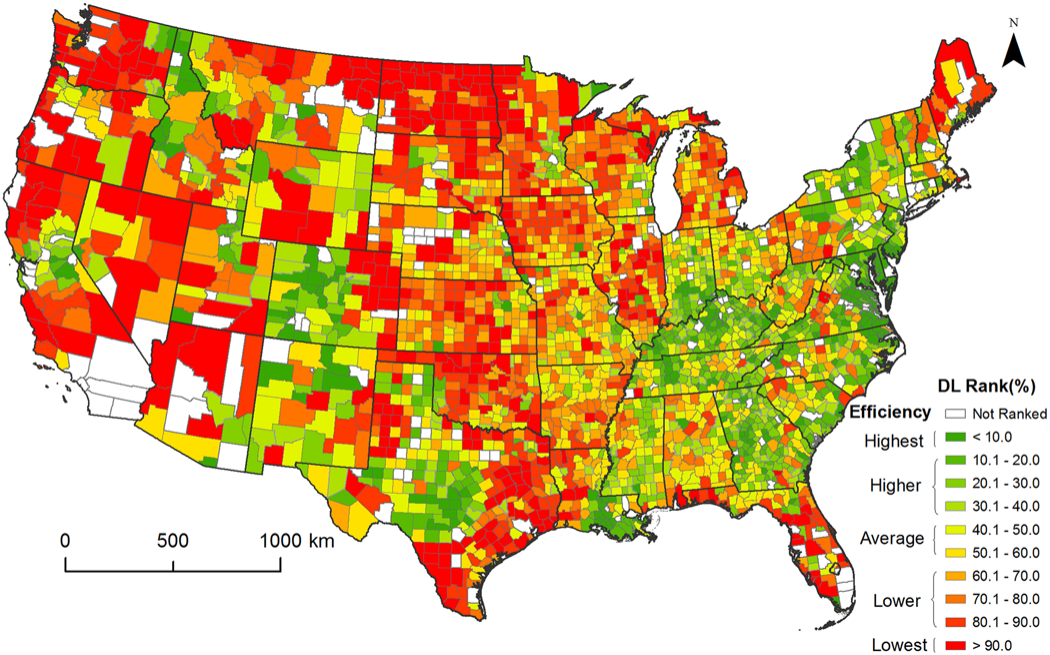
County relative DL consumption ranking contrasted with 100 counties of similar population. Ranking is calculated separately for NMSA and MSA counties. Low county ranking indicates higher efficiency in terms of land consumption than the majority of the counties in the specific group. Ranking close to 50 indicates average DL consumption, while high ranking suggests lower efficiency than the majority of the counties in the specific group.

To better portray county DL efficiency across the conterminous U.S., the relative ranking of the DL value of the reference county within the associated 101 county group is visualized (Fig. 2, S1 Movie). Higher relative ranking position for the reference county reflects higher than expected DL consumption for similar population. The 10^th^/90^th^ DL percentiles depicted in Fig. 1A and Fig. 1B correspond to most and least efficient counties (dark green/red in Fig. 2), while counties close to the median DL values correspond to an average efficiency.

**Fig 3:**
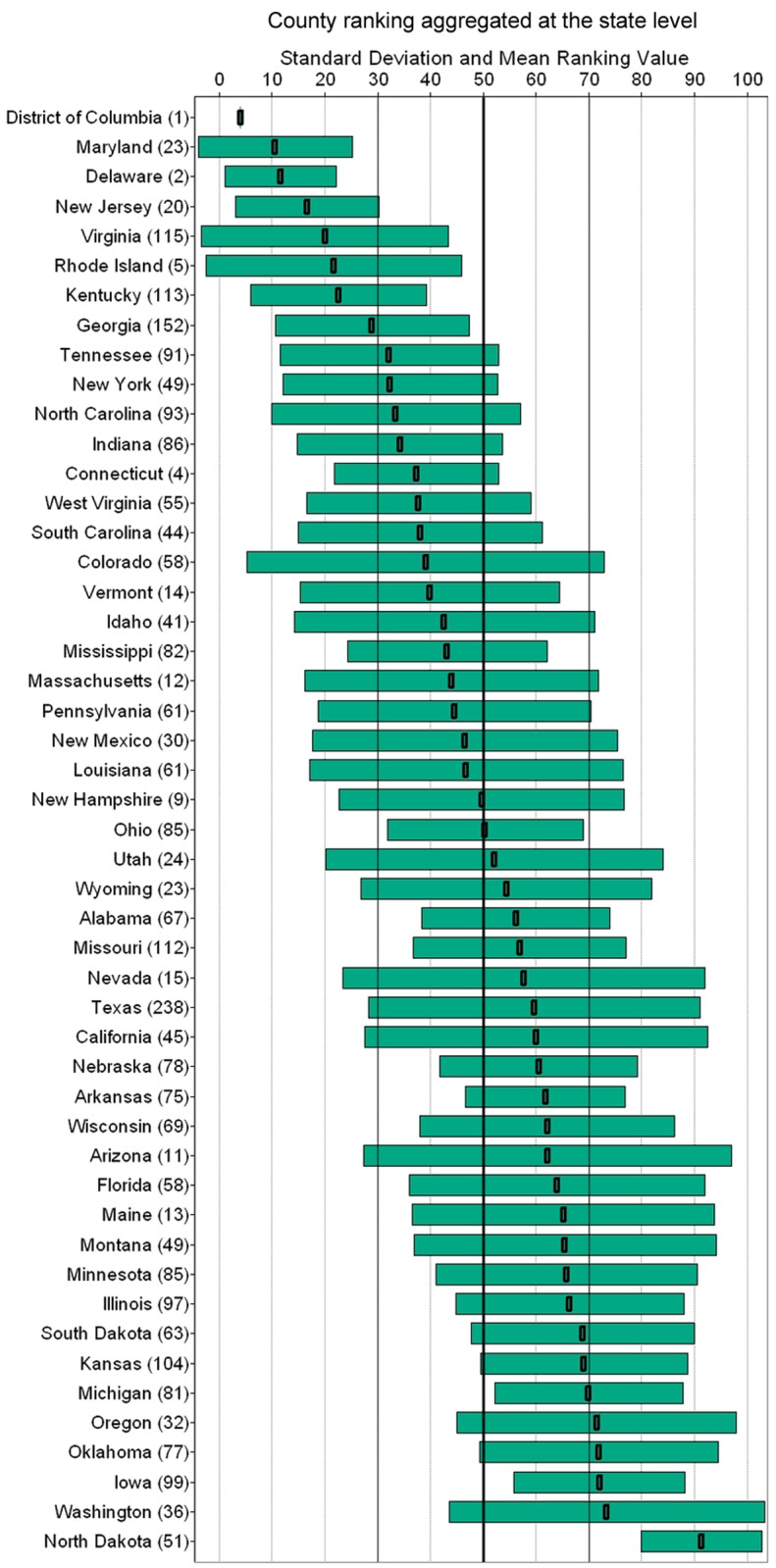
DL consumption county ranking aggregated at state level. Mean ranking value and standard deviation for each state. County total number for each state provided in parentheses. Higher ranking values indicate lower efficiency (for example Oregon, Iowa, Washington and North Dakota). Large standard deviation values reveal high heterogeneity in DL consumption inside the state under examination (e.g. Washington, Arizona and Colorado).

There is a general trend of increased efficiency in the eastern U.S. relative to the rest of the country, partially a result of different historical urbanization patterns, regulations and land availability/suitability. Significant variability exists within states along the Rocky Mountains (Colorado, Montana, Wyoming, Idaho), a contrast to the consistent behavior of states along the Appalachian Mountains. High inefficiency is observed in the agricultural Midwestern states. Surprising differences are present in adjacent states such as Illinois and Indiana. North Dakota is by a large margin the least efficient state in the continental U.S. Complete state comparison is presented in Fig. 3.

### Socioeconomic analysis

**Fig 4:**
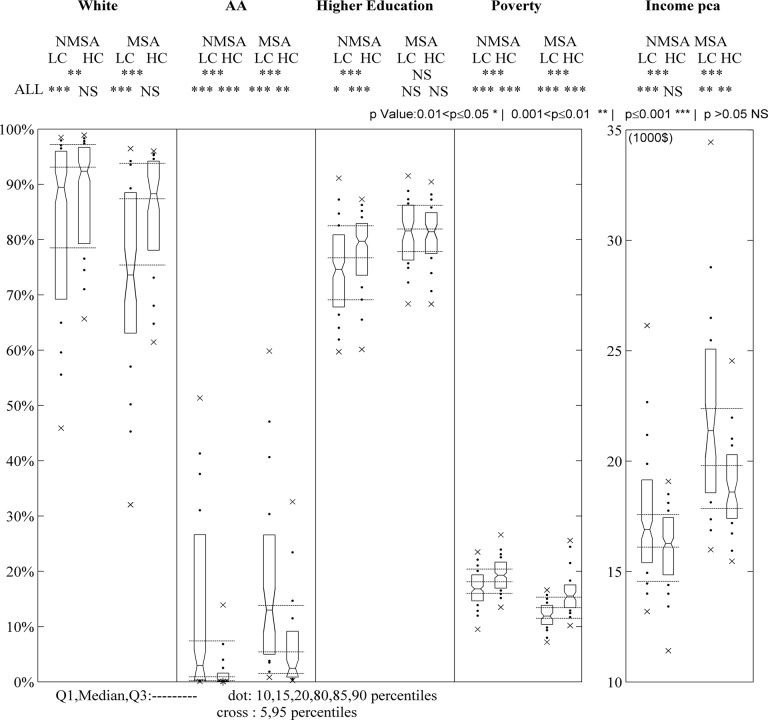
Comparison of socioeconomic characteristics of LC, HC and ALL counties in either MSA or NMSA groups. Each boxplot includes median, 25^th^ and 75^th^ percentiles (marked by upper and lower edges of boxes), 5^th^ and 95^th^ percentiles (cross symbol) and 10^th^, 15^th^, 20^th^, 80^th^, 85^th^, 90^th^ percentiles (dot symbol). Horizontal dashed lines express median, 25^th^ and 75^th^ percentiles for the MSA and NMSA total group (S2 Table). The Mann-Whitney U-test results show statistically significant differences between LC/ALL, HC/ALL and LC/HC counties for most SE characteristics (S3 Table, Text C in S1 File).

DL low consumption (LC) MSA/NMSA counties when compared with all MSA/NMSA counties respectively have strong racial composition with significantly lower White and higher AA populations (Fig. 4). Furthermore, Education levels are lower for NMSA LC counties when compared to ALL NMSA and show no significant difference in MSA counties. LC counties exhibit better economic conditions with lower Poverty and higher Income levels, suggesting that more affluent counties are more efficient with their DL footprint ([56,2]). This contradicts the typical race and education correlations with economic variables (Whites tend to have positive correlation with income while AA negative, Higher Education level is associated with higher Income), especially for NMSA counties (Text B in S1 File).

DL high consumption (HC) MSA/NMSA counties when compared with ALL MSA/NMSA counties have lower AA population rates but no significant differences in White composition. This is interesting as AA and Whites have strong correlation by design. Poverty rates are significantly higher in HC counties. Further analysis indicates that Poverty rates offer the strongest association with DL consumption compared to other SE metrics (S6 Fig., S7 Fig.). This may be attributed to the fact that poverty, which is often associated with high unemployment rates [57], may induce population decreases through high migration rate out of the area to pursue employment. This migration pattern leads to high concentration of vacant housing and underutilized infrastructure (e.g. dwellings, parking lots, commercial buildings) [58] and as a result an increase in per capita DL consumption. Income is only statistically lower in HC MSA counties when compared with ALL MSA counties but there is no statistical difference for HC NMSA with ALL NMSA. Education is higher in HC NMSA counties but shows no statistical difference in MSA counties, reinforcing the observation that Education matters in DL consumption for NMSA counties but not for MSA ones.

Comparing at the overall NMSA- MSA level (median values), MSA have higher AA population, and higher level of education comparing to NMSA. In addition NMSA counties have far smaller income pca than MSA counties as well as higher poverty. The relations of economic growth and poverty within urban environment and the relation of higher income with more populated areas have also been reported by many studies ([56],[57]).

Guided by county and state DL efficiency ranking, policy makers can now identify areas of over-consumption for further mitigation planning. Successful containment policies and/or smart growth strategies from the highly efficient areas could offer examples of proven mitigation strategies, thus advancing a more sustainable DL future consumption. The developed land consumption patterns also identify specific socioeconomic groups, thus allowing development of national strategies for more equitable consumption. Although we are not attempting to identify the underlying mechanisms of cause and effect between the SE characteristics and consumption patterns, our DL ranking can be used as the initial screening mechanism for identification of successful policies and later implementation to other counties.

To summarize, in this work we expand beyond the highly populated city boundaries and study the landscape of the entire conterminous U.S. The overall findings reveal four conclusions for policy makers:

1. The power relationship linking DL consumption and population that was previously suggested for highly populated areas does not hold true for the vast majority of the landscape outside city limits. Instead the relationship is mostly linear altering the notion the expected population growth will have lower DL consumption if added in counties with larger population. Our results suggest that added DL consumption is independent of county’s starting population and only dependent whether the county is rural or urban. At the national stage this finding may change widely accepted current policies.
2. For the first time a national comparative guide exists in term of DL consumption efficiency. This allows identification of over- and under-performing counties (and subsequently states) bringing further to the foreground future geographic allocation of expected population growth.
3. There is a reasonable expectation that urban and suburban counties have higher efficiency in terms of DL consumption than rural counties. For the first time we quantify this efficiency gain, for the median values it is a constant value of approximately 20km^2^. The fact that this gain is constant throughout the overlapping population range suggests that transitioning from rural to urban has significantly higher benefits in lower population, in relative terms.
4. DL low consumption counties in both NMSA and MSA are characterized by higher AA population, lower education level, lower poverty, and higher pca income. On the other hand, counties with lower AA population, higher poverty, and lower income pca tend to increase DL consumption in both NMSA and MSA. Higher education, although it is significant in NMSA counties, it is not for MSA counties. In addition to the aforementioned significant findings, there are certain limitations to our study. More socioeconomic variables like annual growth in GDP per capita, capital flows, agricultural vs industrial economy, land use policy and transportation costs are an important future supplement for this work to analyze the complex interactions between socioeconomic characteristics and DL. In addition, this study includes one timestamp; it would be interesting to track DL dynamics over time. Still, by using space for time substitution as referred earlier we can assume a temporal trend by studying counties of different population. Finally, we concentrate our analysis only to median DL values. Although there is variability in DL values, especially in MSA counties, we focus on median values in order to build models linking DL area to population and thus draw initial conclusions on the relationship between a county's population and its DL footprint. In the future, variability could be further analyzed to reveal potential hidden structure in the data.

The projections for intense upcoming expansion of urban areas worldwide is estimated to cause significant land cover changes posing serious environmental challenges that will spread well beyond the vicinity of cities [1,2,5]. The significance of these consequences has led the United Nations to rank managing urban growth as one of the most important challenges of the 21st century [20]. The fact that urban land is expected to double worldwide by 2030 [1] suggests that policy actions should be taken to shape a sustainable future. The urgency of incorporating sustainable settlement growth into strategic urban planning has recently increased [59]. Still, globalization combined with the looming land scarcity significantly complicates future pathways for policy actions on land resource savings [16] and smart urban growth and compact cites have recently received attention as urban planning initiatives [60]. A balance should be found between creating compact environments that lower DL consumption levels while taking into account urban and rural ecosystem changes [61] and considering the negative implications of higher urban densities, including crime, congestion, extreme microclimates and pollution as well as the environmental changes affecting non urban areas as for example the transition of forest to developed land [61]. The road towards environmentally efficient and sustainable land use/cover policy presupposes better understanding of land consumption patterns and trends. Our work adds important insights towards policies for a sustainable future by establishing DL efficiency standards and expected consumption models.

## Supporting Information

S1 Fig DL ranking map overlaid with the 10 geographic regions to calculate the thematic accuracy of NLCD 2001. National accuracy results have been assessed on 10 geographic regions with user’s and producer’s accuracy for DL class (Level I, NLCD 2001 Class 20, mode definition) ranging from 57.0–92.9% and 43.0–87.0%, respectively (Reference 7 in S1 File).
(TIF)

S2 Fig Metropolitan Statistical Areas (MSA) and Non Metropolitan Statistical Areas (NMSA) counties. Excluded counties (200 total) cannot form symmetrical groups based on having 50 prior or 50 subsequent counties during the population ranking process. They are regarded as population outliers.
(TIF)

S3 Fig DL, population scatter plots for NMSA and MSA counties. There is a moderate correlation coefficient in NMSA counties (r = 0.55) and a strong correlation for MSA counties (r = 0.80). There is high variability in DL values for similar populations. For example, for a NMSA county with population of 10,000 people, the DL value varies between 8.3 km^2^ and 222km^2^. For this reason a locally adjusted population ranking is produced by comparing each county’s DL to others of similar population (50 with the closest yet smaller population than the reference county and 50 with closest and larger population).
(TIF)

S4 Fig Developed Land (DL) for the conterminous U.S. calculated using satellite-derived data from the National Land Cover Database 2001 at a 30m cell size. Counties with large values in DL can be found mainly round Metropolitan Statistical Areas in California, in Middle Atlantic, in New England and in northern parts of South Atlantic division. On the other hand counties with small DL can be bound in more rural areas in Mountain and Mid-West divisions.
(TIF)

S5 Fig MSA and NSMA counties inside the overlapping population range (23,354–91,292 people). A 35.7% of all examined NMSA counties falls inside this range (775/2167) and 38.3% of all examined MSA counties (284/742).
(TIF)

S6 Fig Comparison of socioeconomic characteristics of NMSA counties per 10 ranking positions, and ALL NMSA counties. Each boxplot includes median, 25th and 75th percentiles (marked by upper and lower edges of boxes). Horizontal dashed lines express median, 25th and 75th percentiles for the NMSA ALL group per SE. The Mann-Whitney U-test results show statistically significant differences between some groups and ALL counties. There seems to be an increasing pattern in Higher Education (with the first ranking group-LC exemption). Last three groups have similar results. Poverty first two and last two groups show statistical significant differences with ALL counties, while intermediate groups are similar. AA population presence is more evident in first three ranking groups. Income pca is only differentiated along the first ranking group (LC). *: 0.01<p≤0.05, **: 0.001<p≤0.01, ***: p≤0.001, NS: p>0.05 (See S4 Table for p-values).
(TIF)

S7 Fig Comparison of socioeconomic characteristics of MSA counties per 10 ranking positions, and ALL MSA counties. Each boxplot includes median, 25th and 75th percentiles (marked by upper and lower edges of boxes). Horizontal dashed lines express median, 25th and 75th percentiles for the NMSA ALL group per SE characteristic. Poverty has a strong increasing pattern. income pca has (with the exception of HC/LC groups) not statistical significant differences with ALL counties White population in the first ranking group (LC) is the only that is statistical different than ALL counties. Higher education has an up and down pattern showing no statistical significant difference at any raking group, with ALL counties. *: 0.01<p≤0.05, **: 0.001<p≤0.01, ***: p≤0.001, NS: p>0.05 (See S4 Table for p-values).
(TIF)

S1 Table Modal User's and Producer's accuracy for DL for the 10 regions used for the thematic accuracy assessment of the NLCD 2001 land cover for the conterminous U.S. A consistent overestimation of DL throughout the 10 geographic regions, with the exception of region 4, where DL is underestimated.
(PDF)

S2 Table 75th percentile, median, mean, 25th percentile and standard deviation values for NMSA and MSA counties for the five SE characteristics. (PDF)

S3 Table P- values for the Mann-Whitney U-Test for NMSA and MSA counties per SE characteristic, consumption group (LC/HC) and ALL counties. (PDF)

S4 Table P- values for the Mann-Whitney U-Test for NMSA and MSA counties per SE characteristic, 10 ranking groups and ALL counties. (PDF)

S5 Table Pearson’s correlation coefficient. White and AA have strong negative correlations. All other variables have weak correlations with the exception of income and higher education that have moderate correlations (r = 0.62 in NMSA and r = 0.66 in MSA). In MSA higher education has a moderate to weak correlation with poverty (r = 0.54). P-value is the probability of getting a correlation as large as the observed value by random chance, when the correlation is zero (null hypothesis). All correlations are statistical significant at the p<0.01 level. Asterisk (*) indicates correlations not significant at this level.
(PDF)

S1 Movie Further investigation on individual counties. Each movie frame is associated with one of the 3109 counties (2909 reference counties and 200 excluded counties). For each reference county the +/-50 closest population counties are depicted along with the ranking position (efficiency) of the reference county inside the 101 group, the DL in km2, the population, the DL per capita and the median DL value in km2. The reference county is highlighted with a red circle. It is also indicated whether it is an MSA or NMSA county as well as the state it belongs to. The movie frames are sorted in ascending order by state and then by county name. Excluded counties from our analysis are also depicted but with no additional information.
(DOCX)

S1 File (DOCX)
