# Supplementary figures and images for: Sustainable Development under Population Pressure: Lessons from Developed Land Consumption in the Conterminous U.S
Source: PLoS One. 2015 Mar 25;10(3):e0119675. doi: 10.1371/journal.pone.0119675 (PMC4373912; doi:10.1371/journal.pone.0119675)

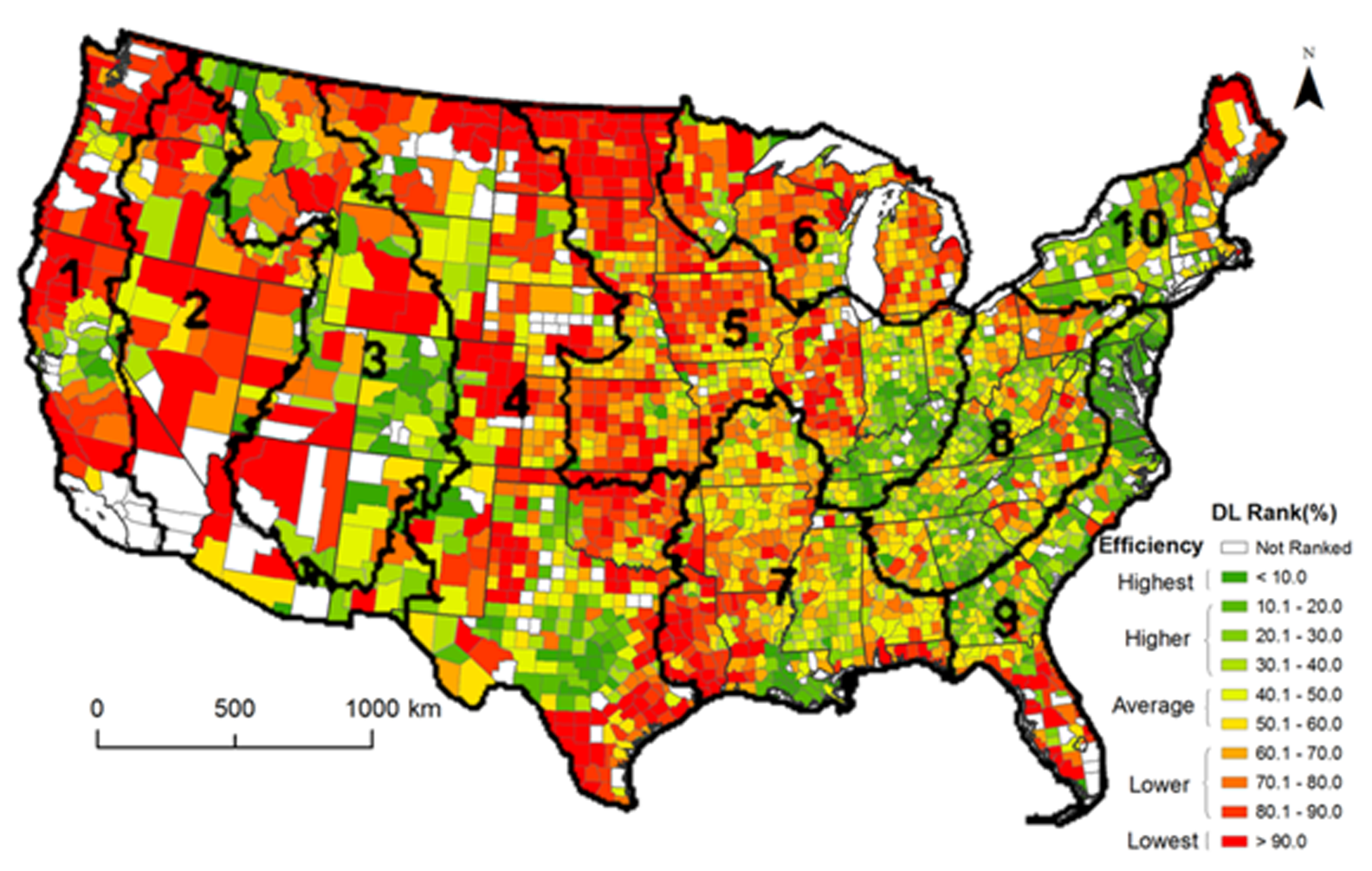

Supplement: S1 Fig — National accuracy results have been assessed on 10 geographic regions with user’s and producer’s accuracy for DL class (Level I, NLCD 2001 Class 20, mode definition) ranging from 57.0–92.9% and 43.0–87.0%, respectively (Reference 7 in S1 File). (TIF) [file pone.0119675.s001.tif]

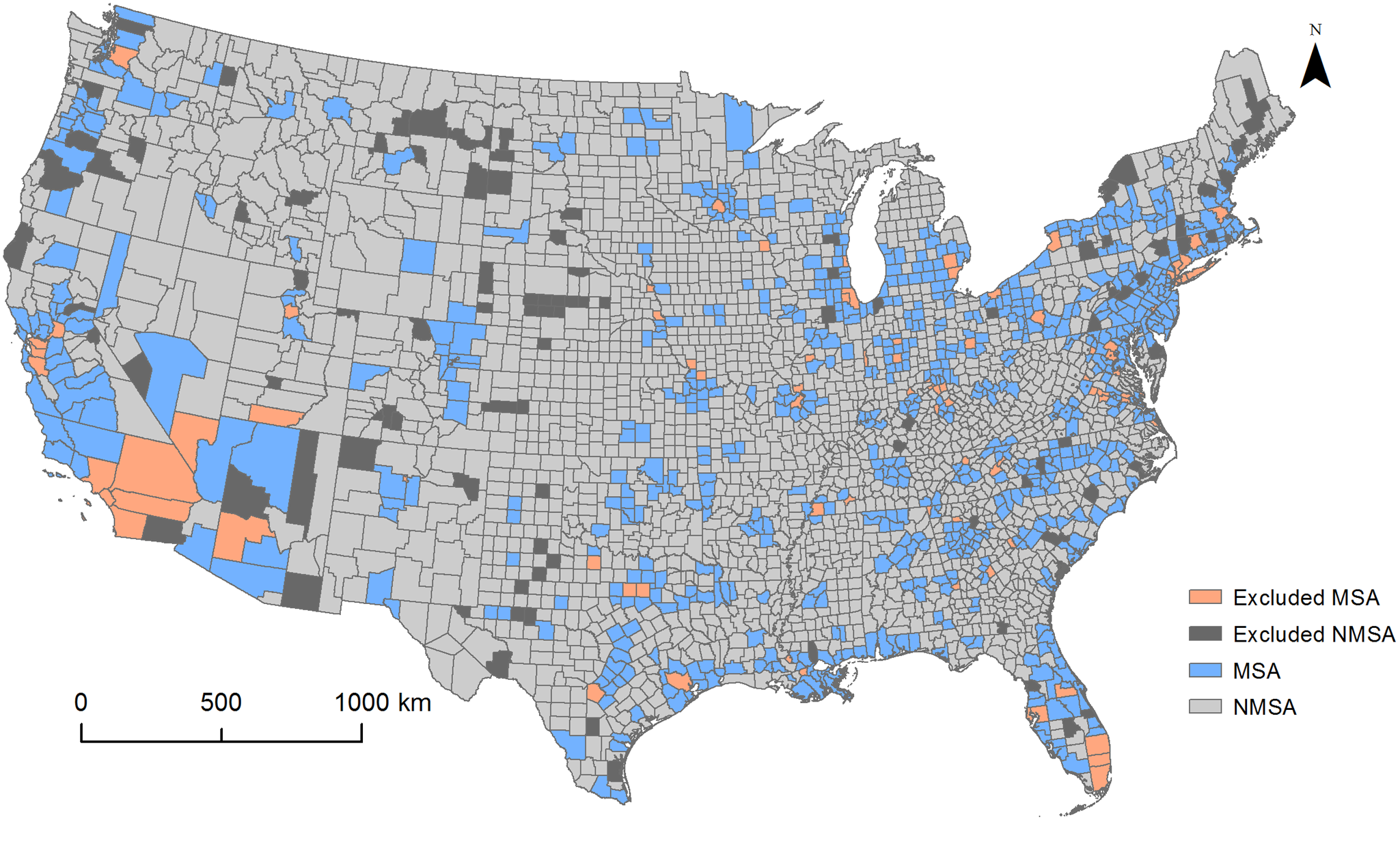

Supplement: S2 Fig — Excluded counties (200 total) cannot form symmetrical groups based on having 50 prior or 50 subsequent counties during the population ranking process. They are regarded as population outliers. (TIF) [file pone.0119675.s002.tif]

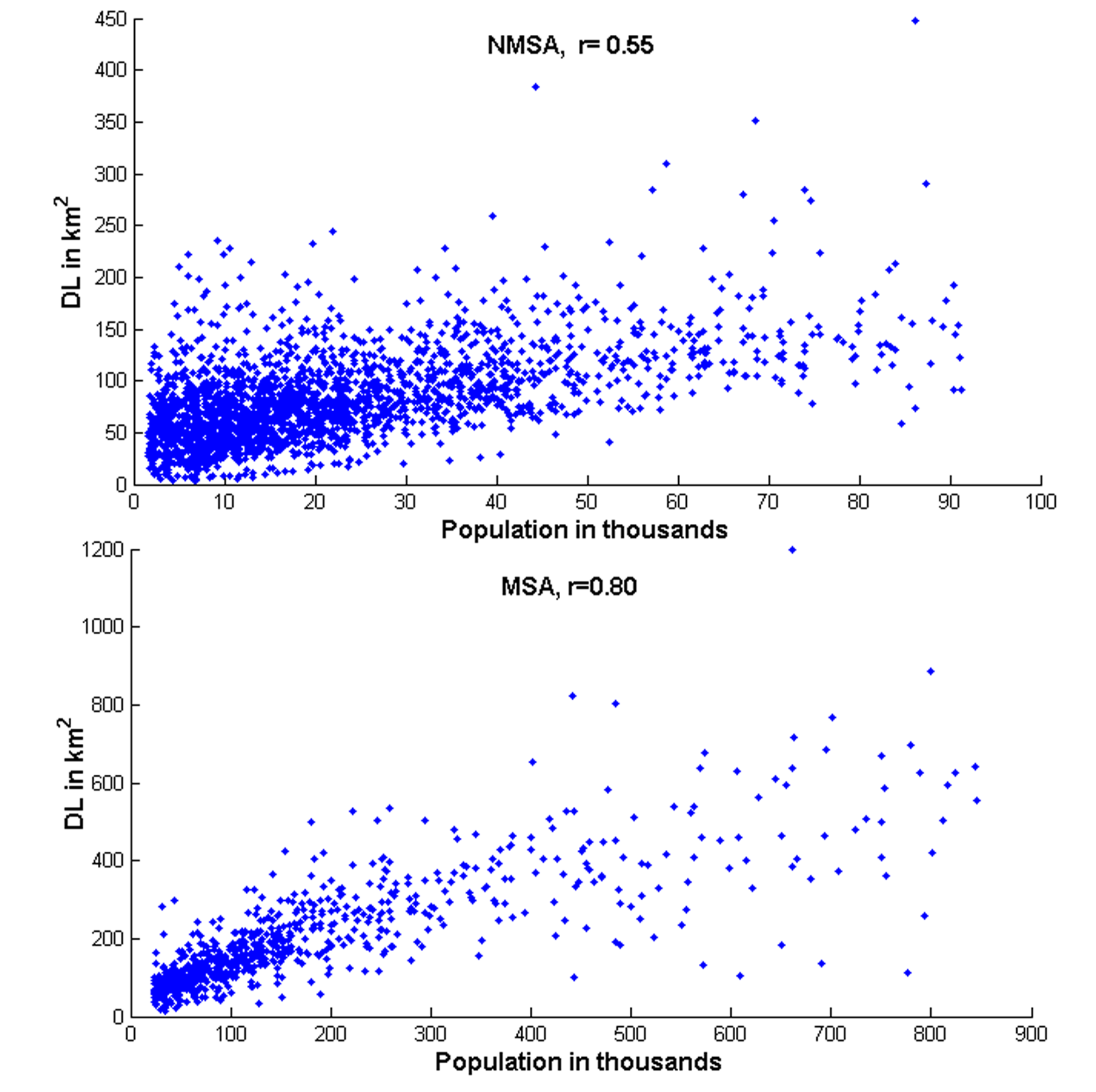

Supplement: S3 Fig — There is a moderate correlation coefficient in NMSA counties (r = 0.55) and a strong correlation for MSA counties (r = 0.80). There is high variability in DL values for similar populations. For example, for a NMSA county with population of 10,000 people, the DL value varies between 8.3 km2 and 222km2. For this reason a locally adjusted population ranking is produced by comparing each county’s DL to others of similar population (50 with the closest yet smaller population than the reference county and 50 with closest and larger population). (TIF) [file pone.0119675.s003.tif]

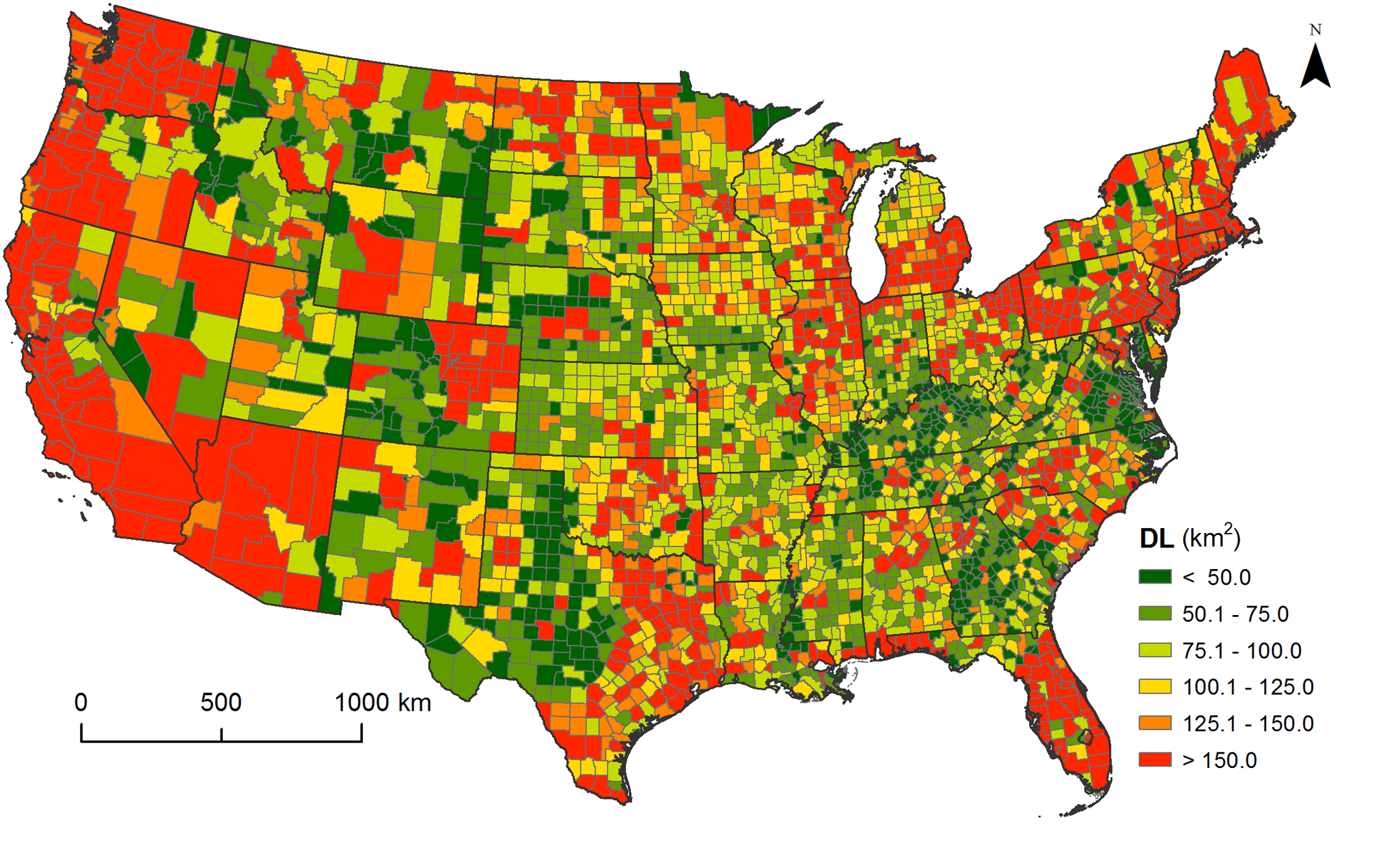

Supplement: S4 Fig — Counties with large values in DL can be found mainly round Metropolitan Statistical Areas in California, in Middle Atlantic, in New England and in northern parts of South Atlantic division. On the other hand counties with small DL can be bound in more rural areas in Mountain and Mid-West divisions. (TIF) [file pone.0119675.s004.tif]

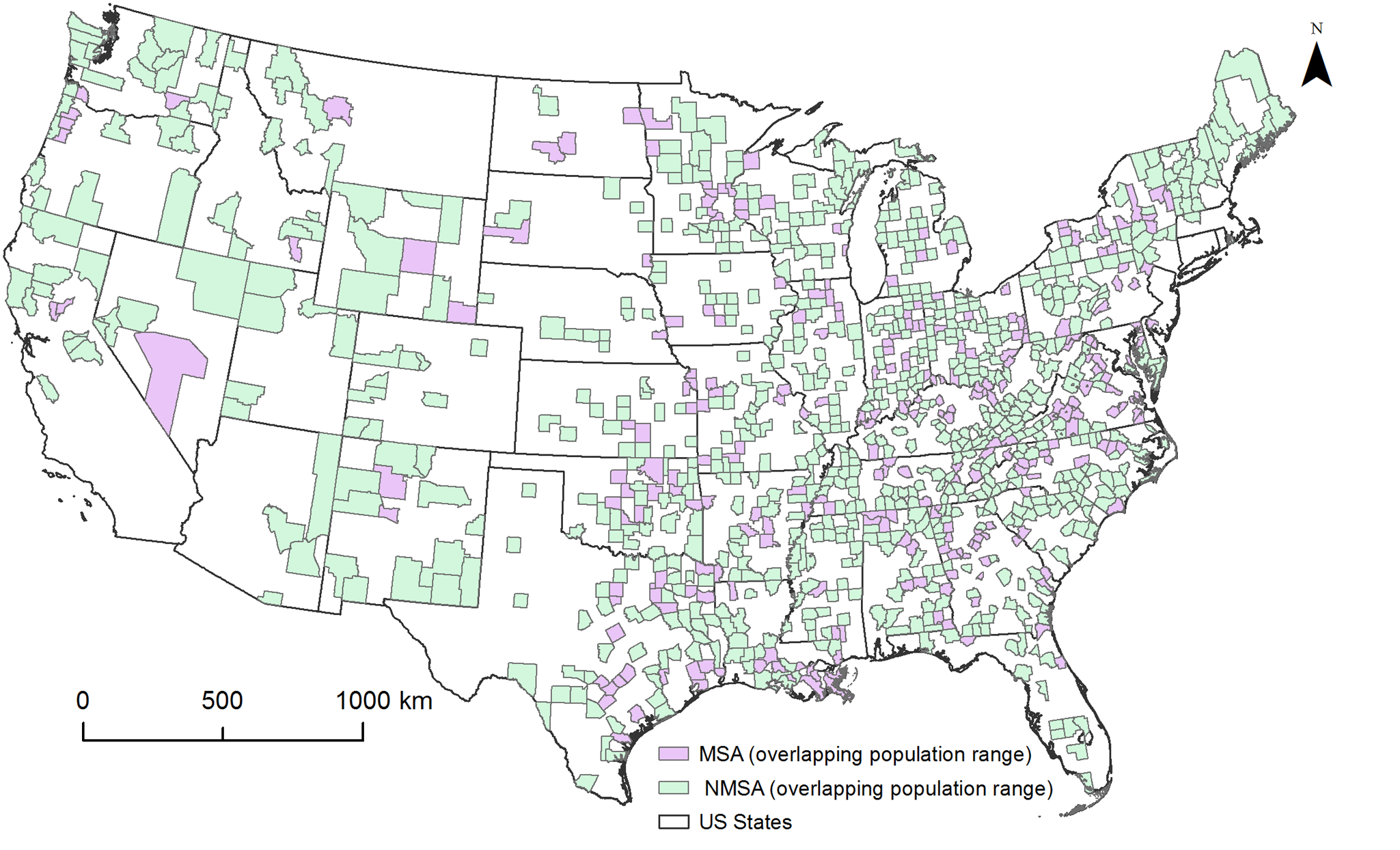

Supplement: S5 Fig — A 35.7% of all examined NMSA counties falls inside this range (775/2167) and 38.3% of all examined MSA counties (284/742). (TIF) [file pone.0119675.s005.tif]

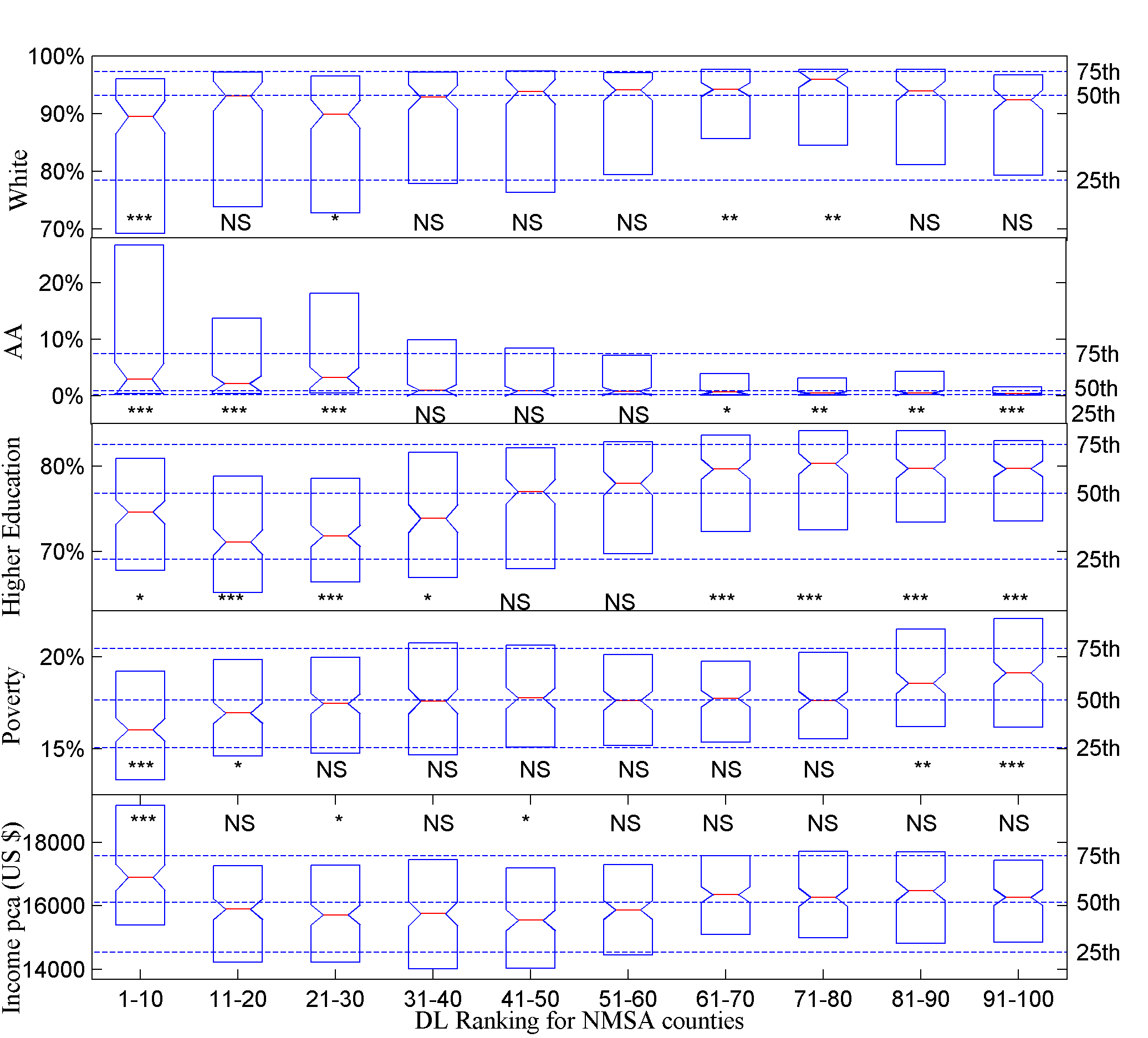

Supplement: S6 Fig — Each boxplot includes median, 25th and 75th percentiles (marked by upper and lower edges of boxes). Horizontal dashed lines express median, 25th and 75th percentiles for the NMSA ALL group per SE. The Mann-Whitney U-test results show statistically significant differences between some groups and ALL counties. There seems to be an increasing pattern in Higher Education (with the first ranking group-LC exemption). Last three groups have similar results. Poverty first two and last two groups show statistical significant differences with ALL counties, while intermediate groups are similar. AA population presence is more evident in first three ranking groups. Income pca is only differentiated along the first ranking group (LC). *: 0.01<p≤0.05, **: 0.001<p≤0.01, ***: p≤0.001, NS: p>0.05 (See S4 Table for p-values). (TIF) [file pone.0119675.s006.tif]

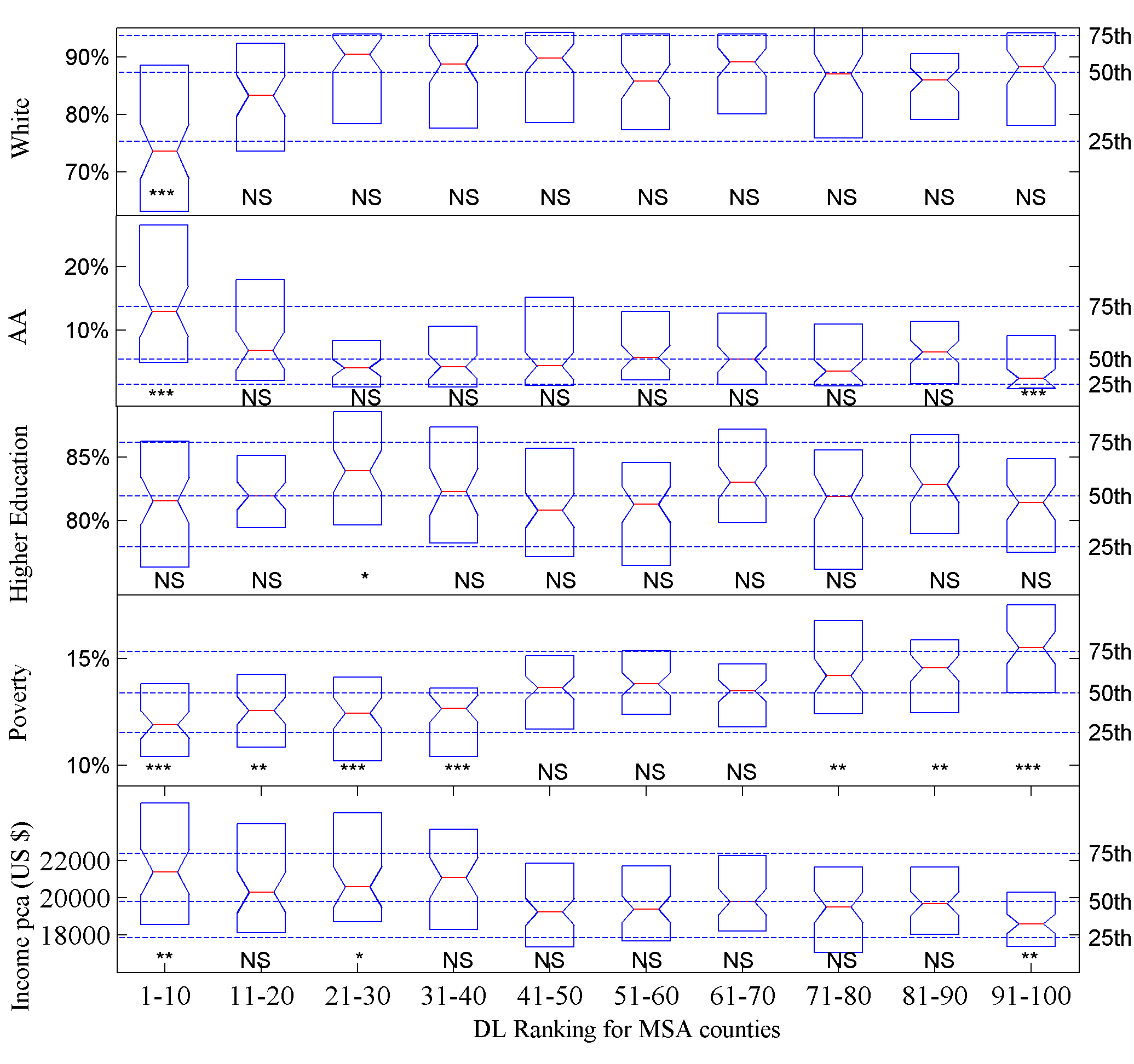

Supplement: S7 Fig — Each boxplot includes median, 25th and 75th percentiles (marked by upper and lower edges of boxes). Horizontal dashed lines express median, 25th and 75th percentiles for the NMSA ALL group per SE characteristic. Poverty has a strong increasing pattern. income pca has (with the exception of HC/LC groups) not statistical significant differences with ALL counties White population in the first ranking group (LC) is the only that is statistical different than ALL counties. Higher education has an up and down pattern showing no statistical significant difference at any raking group, with ALL counties. *: 0.01<p≤0.05, **: 0.001<p≤0.01, ***: p≤0.001, NS: p>0.05 (See S4 Table for p-values). (TIF) [file pone.0119675.s007.tif]
