# Supplementary material for: Sustainable Development under Population Pressure: Lessons from Developed Land Consumption in the Conterminous U.S
Source: PLoS One. 2015 Mar 25;10(3):e0119675. doi: 10.1371/journal.pone.0119675 (PMC4373912; doi:10.1371/journal.pone.0119675)
Supplement: S1 Table — A consistent overestimation of DL throughout the 10 geographic regions, with the exception of region 4, where DL is underestimated. (PDF) [file pone.0119675.s008.pdf]

Table S1. Modal User's and Producer's accuracy for DL for the 10 regions used for the thematic accuracy assessment of the NLCD 2001 land cover for the conterminous U.S. A consistent overestimation of DL throughout the 10 geographic regions, with the exception of region 4, where DL is underestimated.

Regions	1	2	3	4	5	6	7	8	9	10
User's Accuracy	82.9	70.0	76.8	57.0	70.7	92.9	81.6	89.7	92.9	87.0
Producer's Accuracy	70.0	43.0	56.3	87.0	76.8	69.7	61.5	74.3	67.6	62.0
