# Supplementary material for: Sustainable Development under Population Pressure: Lessons from Developed Land Consumption in the Conterminous U.S
Source: PLoS One. 2015 Mar 25;10(3):e0119675. doi: 10.1371/journal.pone.0119675 (PMC4373912; doi:10.1371/journal.pone.0119675)
Supplement: S2 Table — (PDF) [file pone.0119675.s009.pdf]

Table S2. 75th percentile, median, mean, 25th percentile and standard deviation values for NMSA and MSA counties for the five SE characteristics.

SE	NMSA/MSA	Group	75th	Median	Mean	25th	StD
White (%)	NMSA	ALL	97.2	93.1	85.5	78.5	16.6
		LC	96.0	89.5	81.7	69.3	17.8
		HC	96.7	92.4	87.4	79.3	12.4
	MSA	ALL	93.8	87.4	82.9	75.4	13.8
		LC	88.5	73.6	72.6	63.9	19.1
		HC	94.2	88.3	84.1	78.6	12.1
AA (%)	NMSA	ALL	7.4	0.9	8.4	0.2	15.3
		LC	26.6	3.0	13.4	0.3	17.7
		HC	1.6	0.3	2.2	0.1	4.7
	MSA	ALL	13.8	5.4	10.1	1.5	12.5
		LC	26.4	13.0	19.1	5.4	18.2
		HC	8.9	2.5	7.2	0.9	10.2
Higher Education (%)	NMSA	ALL	82.5	76.7	75.5	69.1	8.8
		LC	80.9	74.6	74.4	67.9	9.4
		HC	82.9	79.7	77.3	73.6	9.0
	MSA	ALL	86.2	81.9	81.4	77.8	6.5
		LC	86.1	81.6	81.1	76.4	6.6
		HC	84.7	81.4	80.3	77.5	6.9
Poverty (%)	NMSA	ALL	20.4	18.1	18.3	16.0	3.6
		LC	19.4	16.8	16.8	14.6	4.2
		HC	21.6	19.3	19.6	16.9	4.2
	MSA	ALL	15.3	13.4	13.7	11.5	3.5
		LC	13.7	11.9	12.0	10.4	2.8
		HC	17.5	15.5	16.4	13.4	4.8
Income (US $)	NMSA	ALL	17584	16105	16188	14552	2761
		LC	19151	16900	17795	15407	4177
		HC	17441	16272	16008	14854	2280
	MSA	ALL	22388	19797	20592	17858	4174
		LC	25032	21384	22411	18598	5194
		HC	20233	18605	19047	17432	2977
