# Supplementary material for: Sustainable Development under Population Pressure: Lessons from Developed Land Consumption in the Conterminous U.S
Source: PLoS One. 2015 Mar 25;10(3):e0119675. doi: 10.1371/journal.pone.0119675 (PMC4373912; doi:10.1371/journal.pone.0119675)
Supplement: S3 Table — (PDF) [file pone.0119675.s010.pdf]

Table S3. P- values for the Mann-Whitney U-Test for NMSA and MSA counties per SE characteristic, consumption group (LC/HC) and ALL counties.

P-values	White (%)		AA (%)		Higher Education (%)		Poverty (%)		Income (US %)	
	NMSA	MSA	NMSA	MSA	NMSA	MSA	NMSA	MSA	NMSA	MSA
LC-HC	0.0021	0.0002	^	^	^	0.6412	^	^	^	^
LC-ALL	^	^	^	^	0.0301	0.6271	^	^	^	0.0022
HC-ALL	0.8756	0.7152	^	0.0091	0.0004	0.2141	^	^	0.8625	0.0013

Symbol ^ indicates values smaller than 10^{-4}.
