# Supplementary material for: Sustainable Development under Population Pressure: Lessons from Developed Land Consumption in the Conterminous U.S
Source: PLoS One. 2015 Mar 25;10(3):e0119675. doi: 10.1371/journal.pone.0119675 (PMC4373912; doi:10.1371/journal.pone.0119675)
Supplement: S4 Table — (PDF) [file pone.0119675.s011.pdf]

Table S4. P- values for the Mann-Whitney U-Test for NMSA and MSA counties per SE characteristic, 10 ranking groups and ALL counties.

NMSA										
Ranking→	1-10	21-30	21-30	31-40	41-50	51-60	61-70	71-80	81-90	91-100
White	^	0.4834	0.0153	0.5501	0.9808	0.8737	0.0049	0.0021	0.0702	0.8733
AA	^	0.0001	^	0.6273	0.9203	^	0.0287	0.0056	0.0077	^
H.Education	0,0304	^	^	0.0339	0.4359	0.4798	0.0003	^	^	0.0004
Poverty	^	0.0353	0.2701	0.9882	0.9419	^	0.7847	0.6739	0.0015	^
Income	^	0.1052	0.0286	0.0730	0.0134	^	0.1594	0.0982	0.4275	0.8686

MSA										
Ranking→	1-10	21-30	31-40	41-50	51-60	61-70	71-80	81-90	91-100	91-100
White	^	0.3210	0.2234	0.6048	0.2328	0.8340	0.1204	0.3283	0.5021	0.7150
AA	^	0.1058	^	0.2839	0.5502	^	0.7846	0.2421	0.9190	0.0088
H.Education	0,6276	^	^	0.5638	0.2908	0.1504	0.0813	^	^	0.2142
Poverty	^	0.0185	0.0024	0.0034	0.7443	^	0.7353	0.0091	0.0034	^
Income	^	0.2147	0.0245	0.1568	0.1354	^	0.6007	0.0988	0.4814	0.0012

Symbol ^ indicates values smaller than 10^{-4}.
