# Supplementary material for: Sustainable Development under Population Pressure: Lessons from Developed Land Consumption in the Conterminous U.S
Source: PLoS One. 2015 Mar 25;10(3):e0119675. doi: 10.1371/journal.pone.0119675 (PMC4373912; doi:10.1371/journal.pone.0119675)
Supplement: S5 Table — White and AA have strong negative correlations. All other variables have weak correlations with the exception of income and higher education that have moderate correlations (r = 0.62 in NMSA and r = 0.66 in MSA). In MSA higher education has a moderate to weak correlation with poverty (r = 0.54). P-value is the probability of getting a correlation as large as the observed value by random chance, when the correlation is zero (null hypothesis). All correlations are statistical significant at the p<0.01 level. Asterisk (*) indicates correlations not significant at this level. (PDF) [file pone.0119675.s012.pdf]

Table S5. Pearson's correlation coefficient. White and AA have strong negative correlations. All other variables have weak correlations with the exception of income and higher education that have moderate correlations (r=0.62 in NMSA and r=0.66 in MSA). In MSA higher education has a moderate to weak correlation with poverty (r=0.54). P-value is the probability of getting a correlation as large as the observed value by random chance, when the correlation is zero (null hypothesis). All correlations are statistical significant at the p<0.01 level. Asterisk (*) indicates correlations not significant at this level.

**NMSA**	White	AA	Higher Education	Poverty	Income
White	1	-0.83	0.47	-0.06*	0.42
Black		1	-0.46	-0.04*	-0.28
Higher Education			1	-0.37	0.62
Poverty				1	-0.51
Income					1
**MSA**	White	AA	Higher Education	Poverty	Income
White	1	-0.86	0.35	0.04*	0.11
Black		1	-0.28	-0.08*	-0.13
Higher Education			1	-0.55	0.66
Poverty				1	-0.47
Income					1
