# Supplementary material for: Sustainable Development under Population Pressure: Lessons from Developed Land Consumption in the Conterminous U.S
Source: PLoS One. 2015 Mar 25;10(3):e0119675. doi: 10.1371/journal.pone.0119675 (PMC4373912; doi:10.1371/journal.pone.0119675)
Supplement: S1 File — (DOCX) [file pone.0119675.s014.docx]

**Supporting Information**

**Text A. Space for time substitution.** Space for time substitution has been highly criticized ranging from rejection (1) to high support (2). It is mostly used when general trends are investigated or when hypotheses are to be generated (3). Still when the past has strong unrecognized effects, the method is not very accurate (3). Although this technique is debated, many studies continue to rely on space for time substitution due to necessity (e.g lack of historical data) or convenience (4, 5, 6). In our case the assumption under the space for time substitution is translated to estimating DL trend from counties of different population. As we are interested for the general trend of efficiency in terms of land consumption and considering our high sample size we believe that this technique will produce reliable results if used.

**Text B. Correlations.** We tested for possible high correlations among SE characteristics. For example, if white population tends to have higher education and AA population tend to have lower education then as whites are gathered more in HC than LC counties, higher education is expected to concentrate on HC counties. The same hypothesis may be addressed for every possible combination of SE. A Pearson’s linear correlation coefficient r is calculated separately for MSA and NMSA counties to discover potential correlations among SE characteristics (Table S5). The only strong (negative) correlation is between whites and AA but this is logical as these values (percentages) are supplementary (high existence of one means low existence of the other). The correlation of white population with higher education is not strong in both MSA and NMSA counties. As a result for the NMSA counties higher education levels is associated with HC counties, irrelevant of the race. Furthermore, the only moderate to weak correlation is between income pca and higher education. Still, although LC NMSA counties tend to have lower values in higher education than all NMSA counties, they have larger values in income pca meaning that their weak correlation does not drive to the same direction DL consumption. All the other SE characteristics have weak correlations and as a result we can not assume that a SE characteristic drives another.

**Text C.** **Comparisons.** Each of the 10 ranking groups is compared with the ALL group for MSA and NMSA respectively using the Mann-Whitney U-Test. First and last groups are the LC and HC (Figure S5, Figure S6, Table S4).

1. Johnson E A, Miyanishi K (2008) Testing the assumptions of chronosequences in succession. Ecology Letters 11:419–431.
2. Walker LR, Wardle DA, Bardgett RD, Clarkson BD (2010) The use of chronosequences in studies of ecological succession and soil development. Journal of Ecology 98:725–736.
3. Pickett STA (1989) Space-for-Time Substitution as an Alternative to Long-Term Studies In *Long-term studies in ecology*, edited by G.E. Likens. Springer Verlag New York 110-135.
4. Chin A (2006) Urban transformation of river landscapes in a global context. Geomorphology 79:460-487.
5. Aragao LE, Shimabukuro YE (2010) The incidence of fire in Amazonian forests with implications for REDD. Science 328:1275-1278.
6. Blois JL, Williams J W, Fitzpatrick MC, Jackson ST, Ferrier S (2013) Space can substitute for time in predicting climate-change effects on biodiversity. Proc Natl Acad Sci USA 110:9374-9379.
7. Wickham JD, Stehman SV,. Fry JA, Smith JH, Homer CG (2010) Thematic accuracy of the NLCD 2001 land cover for the conterminous United States. Remote Sensing of Environment 1286–1296
